# Less water in agriculture? Potential and challenges in optimizing water use efficiency

**DOI:** 10.1093/jxb/erae227

**Published:** 2024-07-10

**Authors:** Emilie Wientjes, Chris Seijger

**Affiliations:** Wageningen University and Research, Laboratory of Biophysics, 6703 WE Wageningen, The Netherlands; Wageningen University and Research, Water Resources Management, 6700 AK Wageningen, The Netherlands

**Keywords:** Crop, drought, food, PsbS, water use efficiency

## Abstract

This article comments on:

Turc B, Sahay S, Haupt J, de Oliveira Santos T, Bai G, Glowacka K. 2024. Up-regulation of non-photochemical quenching improves water use efficiency and reduces whole-plant water consumption under drought in *Nicotiana*  *tabacum*. Journal of Experimental Botany 75, 3959–3972.

This article comments on:

**Turc B, Sahay S, Haupt J, de Oliveira Santos T, Bai G, Glowacka K. 2024. Up-regulation of non-photochemical quenching improves water use efficiency and reduces whole-plant water consumption under drought in *Nicotiana*  *tabacum*. Journal of Experimental Botany 75, 3959–3972.**

**Agriculture is a main user of fresh water. Excess water use can lead to the depletion of groundwater and surface water sources, with detrimental effects for the environment and society. Providing enough food for a growing population in a (water)-sustainable way is one of the major challenges of the 21st century. It can only be tackled by the combination of strategies. One strategy is to develop crops with a higher water use efficiency (WUE). In simple terms ‘more crop per drop’. Turc *et al*. (2024) have shown that up-regulation of non-photochemical quenching (NPQ) reduces water consumption in tobacco plants under drought stress. In this Insight article, we discuss this work in a wider context of approaches and challenges to improve WUE in crops.**

The demand for fresh water in agriculture is expected to rise further in the coming decades given that the world population is growing, diets change, and the temperature increases. Excess water use has several negative consequences. For instance, depletion of groundwater and surface water resources impacts the availability of water for ecosystems in the surroundings and leads to reduced biodiversity. Excessive agricultural water use can exacerbate water scarcity issues, leading to competition and conflict for water between farmers, industry, households, and the environment. Linked to the competition for fresh water are economic consequences, such as increased costs for water extraction and increased chances of a failing harvest when not enough water is available. A failing harvest increases the risk for widespread food insecurity in regions where people already have limited access to food. It is clear that much is at stake to bring agricultural water use within the planetary boundaries, while at the same time producing enough food to feed the growing world population (Foley *et al*., 2011) and conserving sufficient land and water for nature. This grand challenge requires a multifaceted approach to increasing food production and reducing agriculture’s environmental footprint (Foley *et al*., 2011). The article by Turc *et al*. (2024) investigates the potential to save water by genetic modification of plants so that they become more efficient in their water use.

## WUE in plants and what has been done to improve it

**Fig. 1.**
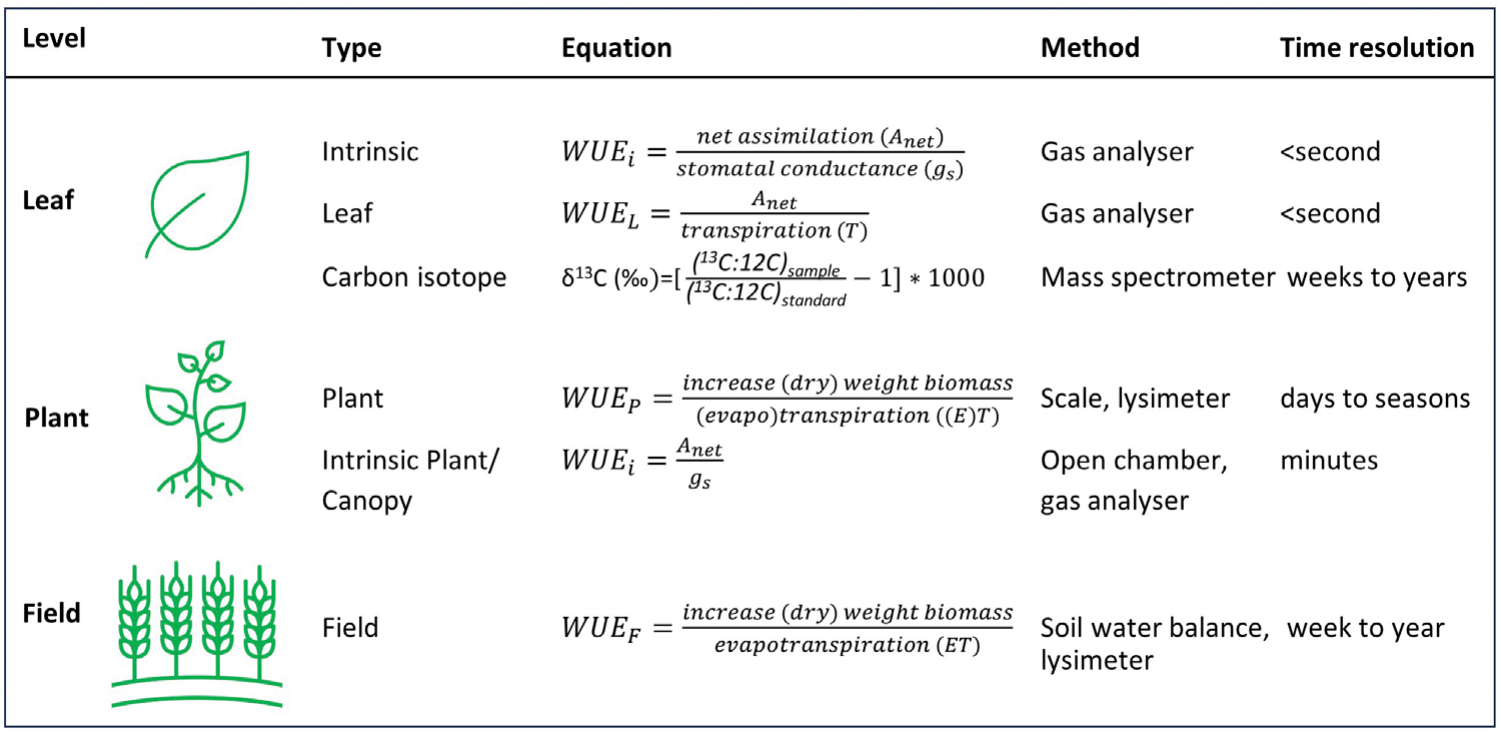
Water use efficiency at different scales. The intrinsic WUE (WUE_i_) can be measured at the leaf, plant, or small canopy level (Leakey *et al*., 2019; Hoover *et al*., 2023). At the leaf level, the WUE_i_ [the net amount of CO_2_ fixed (*A*_net_) per stomatal conductance (*g*_s_)] or the leaf-level net CO_2_ assimilation per water transpiration is reported. Differences in the carbon isotope composition of plant tissue have been used as a high-throughput method for assessing WUE as it correlates well with WUE for C_3_ plants. For the WUE_P_, the increase in (dry) weight of biomass per water use is assessed. For the WUE_F_, the biomass can be measured directly by harvesting crop biomass. Evapotranspiration is measured by a lysimeter or calculated with a soil water balance.

Increasing the WUE of crops (i.e. the amount of carbon assimilated per unit of water lost) could contribute to reduced water use in agriculture. Water loss in plants is unavoidable as stomata at the leaf surface have to open to allow CO_2_ to diffuse inside the leaf, at the same time allowing water vapor to escape. The term WUE has been defined in several ways among different scientific disciplines, and is measured among different time scales and with different methods (Fig. 1). WUE increases with increasing atmospheric CO_2_ concentration and mild drought stress, and it decreases under nitrogen limitation and increasing evaporative demand of the surrounding atmosphere (when air is drier, hotter, and windier) (Steduto *et al*., 2007; Leakey *et al*., 2019). The impact of climate change on WUE of crops is difficult to determine. On the one hand is the positive impact of current rising CO_2_ levels because in one time interval more carbon can be assimilated per unit of water lost. On the other hand is the negative impact of global warming decreasing the WUE when the temperature increases above the optimum temperature for the crop, thereby slowing down carbon fixation (Hatfield and Dold, 2019).

Under identical environmental conditions, the WUE of C_4_ plants (e.g. maize and sorghum) is almost twice as high as that of C_3_ plants (e.g. rice and wheat) (Steduto *et al*., 2007). While leaf-level WUE varies substantially throughout the day, it has been shown that these variations average out over days, and that the WUE at plant level is approximately constant for a given crop species after normalization for the evaporative demand of the atmosphere and air CO_2_ concentration (Steduto *et al*., 2007). After this normalization, there is very limited variation between the WUE of several C_3_ crops under non-stressed field conditions. This conserved relationship, between biomass produced and water used, suggests that there is a limited scope to improve WUE in the field when the environmental conditions such as CO_2_ concentrations and evaporative demand of the atmosphere cannot be changed. On the other hand, during the past century, breeders have made use of the fact that the environmental factors strongly affect the WUE, by generating crops that can grow earlier or later in the season when the weather conditions are more humid and cooler. This approach has led to improved WUE and crop yield (Condon *et al*., 2004).

Currently there are three genetic research approaches that signal potential to improve the WUE of crops further. The first is genetic modification of plants, which is the approach used by Turc *et al*. (2024). Several genetic manipulation studies have demonstrated improved WUE at the intrinsic or plant level (Leakey *et al*., 2019). Many of those have targeted the stomata either directly or indirectly. One example is the overexpression of the PSII subunit S (PsbS) protein (Glowacka *et al*., 2018). PsbS down-regulates the excitation energy flow towards PSII under high light by NPQ, as such protecting the plant against photodamage (Li *et al*., 2000). The increased level of NPQ under high light in the PsbS overexpression lines results in a more oxidized Q_A_ pool compared with wild-type plants. The Q_A_ redox state is linearly correlated with stomatal conductance (*g*_s_). Overexpression of PsbS resulted in a decreased *g*_s_ under high light, that was not matched by a decrease in net CO_2_ assimilation, leading to an increased intrinsic WUE (WUE_i_) (Glowacka *et al*., 2018). Turc *et al*. (2024) follow this approach of using PsbS overexpression. PsbS from *Arabidopsis thaliana* was overexpressed in tobacco plants. In the current study, it is shown that under drought stress (60% field water capacity, FWC) the WUE_i_ increased by ~15% for the transgenic lines under high light. Furthermore, the total water consumption per plant was significantly lower for the PsbS overexpression lines under control (80% FWC) and drought stress (65% FWC) during 6–9 d of the experiment. At the same time, the biomass of the drought-stressed transgenic plants was not significantly reduced compared with the wild type. As the role of PsbS is conserved in all land plants, the overexpression of the protein can have the same positive effect on WUE in crops. Having crops that use less water under control and drought stress conditions would be a great advantage for agriculture and society.

The second approach builds on the insight that the progenitors of crops have evolved under lower CO_2_ levels and temperatures than we currently face, and both are predicted to increase in the near future. This could mean that crop WUE is not optimized for current climatic conditions; in other words, the climatic conditions changed too recently for evolution to adapt to it. Following this observation, Drewry *et al*. (2014) proposed changing the leaf canopy structure to have plants with more vertical leaves. This would allow lower leaves to receive more light and as such carry out more photosynthesis. As the vapor pressure deficit is lower at this position in the canopy, the WUE is predicted to increase (Drewry *et al*., 2014).

The third approach focuses on variation in WUE between genotypes, which has been reported for a number of crop species (Hatfield and Dold, 2019; Leakey *et al*., 2019). This variation can be used for natural breeding for higher WUE. However, there is a potential risk associated with selecting for these genotypes, as they often demonstrate decreased drought resistance and slower biomass production (Steduto *et al*., 2007; Blum, 2009). Nevertheless, this approach has been successful for bread wheat in Australia. By evaluating the stable carbon isotope composition of plant tissue, as proxy for WUE in C_3_ species, wheat varieties were selected that have a better grain yield under drought conditions (Rebetzke *et al*., 2002).

## Carrying WUE gains from the lab to the field

Turc *et al*. (2024) performed experiments on potted tobacco plants grown in a greenhouse for 6–9 d in order to analyze physiological responses under controlled temperature conditions. In the future, evaluation of the WUE_P_ for an entire crop cycle—from planting until harvest—would be of high interest. Furthermore, research is needed to explore if the WUE gains found in controlled greenhouse conditions persist under field conditions. When gains in WUE persist at the field level, the up-regulation of NPQ becomes a promising approach to improve WUE in crops in open agricultural fields.

What is the potential to scale WUE improvements from the lab to the agricultural field? Depending on the growth condition, the PsbS overexpression lines in the study of Turc *et al*. (2024) consumed up to 30% less water than the corresponding wild type. If this would translate to a similar reduction of WUE for crops in the field, the impact would be tremendous. Yet, it can be challenging to translate the increased WUE from the lab to the field, where variables such as temperature, water availability, and nutrient supply are (much) less regulated (Molden and Oweis, 2007). It could be that single gene mutations are favorable under controlled conditions, but are outcompeted by their wild-type counterparts in the outside world. In order to realistically assess the potential of WUE gains, it is crucial to assess how improvements observed at the leaf level translate to the canopy and, subsequently, to broader field conditions and agricultural settings (Digrado *et al*., 2020).

Scaling WUE from the leaf to the open field level necessitates a comprehensive understanding of the physiological factors influencing each level and the mechanistic links between them (Hoover *et al*., 2023). Larger scale field experiments on transgenic plants, such as the PsbS over-expression mutant of Turc *et al*. (2024), contribute to this understanding. Ideally, models would be developed that can accurately predict carbon assimilation and water use under given environmental conditions (e.g. CO_2_ concentration, temperature, water, and nutrient availability) for a given crop genotype that then could be studied in a field experiment. Such models could also help to understand what traits are important to cope well with expected changes in climate, with increased temperatures, increased drought alternated with extreme rain, and increased atmospheric CO_2_ concentrations. Using field-level experiments, a comparison between the transgenic plant and the wild type can then empirically reveal how much of the WUE improvement found in the lab is transferred to the open field (Box 1). We call for interdisciplinary studies that explore how WUE gains observed in the lab translate to the open field to uncover the potential of different genetic research approaches to optimize WUE of crops.

Box 1. **Measuring WUE at the field level**

Laboratory experiments and models serve as indispensable tools in elucidating WUE in plants. In controlled settings, they offer precise manipulation of variables, enabling detailed exploration of plant physiology and responses. Next, field experiments are necessary to test model and lab approaches to increase WUE at the field level and distinguish lab gains from field gains in WUE. The following criteria apply for an accurate measurement of biomass and evapotranspiration in the field. (i) The transgenic plant is compared with a control plant representing current farming practice. The experiment is conducted at one site for one growing season to control for environmental variation. (ii) Above-ground dry biomass is harvested three times to represent different crop growth stages of: crop development (when ground cover increases, before flowering), mid-season (full ground cover, after flowering, before aging), and late season (harvest) (Allen *et al*., 1998). The late season harvest is also used to determine dry weight commercial biomass for both crops. (iii) The experiment is replicated four times to generate reliable data. This means that 24 experimental plots are needed to replicate the treatment (transgenic plant, control plant) in three harvests of biomass (development, mid-season, late season). (iv) Evapotranspiration is measured with a lysimeter or assessed through the soil water balance method where each water balance component is measured and evapotranspiration can then be calculated as the sum of rainfall, irrigation, capillary rise, change in soil moisture content, minus run-off and deep percolation. (v) Within season data on biomass and evapotranspiration are shared for reasons of transparency and verifiability.

Biomass and crop evapotranspiration are linearly related throughout a growing season across scales of labs, agronomic research fields, and agricultural systems (De Wit, 1958; Steduto *et al*., 2007; Seijger *et al*., 2023). A quality check of the field experiment is thus to plot biomass and evapotranspiration data for the different growth stages, and check linearity of the data separately for the transgenic plant and control plant. In addition, a field-level gain in WUE should then be manifested in a higher slope of biomass and evapotranspiration data for the transgenic plant than for the control plant.

## References

[CIT0001] Allen RG, Pereira LS, Raes D, Smith M. 1998. FAO irrigation and drainage paper no. 56. Crop evapotranspiration. Rome: FAO.

[CIT0002] Blum A. 2009. Effective use of water (EUW) and not water-use efficiency (WUE) is the target of crop yield improvement under drought stress. Field Crops Research 112, 119–123.

[CIT0003] Condon AG, Richards RA, Rebetzke GJ, Farquhar GD. 2004. Breeding for high water-use efficiency. Journal of Experimental Botany 55, 2447–2460. 15475373 10.1093/jxb/erh277

[CIT0004] De Wit C. 1958. Transpiration and crop yields. Verslagen van Landbouwkundige Onderzoekingen 64, 1–90.

[CIT0005] Digrado A, Mitchell NG, Montes CM, Dirvanskyte P, Ainsworth EA. 2020. Assessing diversity in canopy architecture, photosynthesis, and water-use efficiency in a cowpea magic population. Food Energy Security 9, e236. 33381299 10.1002/fes3.236 PMC7757253

[CIT0006] Drewry DT, Kumar P, Long SP. 2014. Simultaneous improvement in productivity, water use, and albedo through crop structural modification. Global Change Biology 20, 1955–1967. 24700722 10.1111/gcb.12567

[CIT0007] Foley JA, Ramankutty N, Brauman KA, et al. 2011. Solutions for a cultivated planet. Nature 478, 337–342. 21993620 10.1038/nature10452

[CIT0008] Glowacka K, Kromdijk J, Kucera K, Xie JY, Cavanagh AP, Leonelli L, Leakey ADB, Ort DR, Niyogi KK, Long SP. 2018. Photosystem II subunit S overexpression increases the efficiency of water use in a field-grown crop. Nature Communications 9, 868. 10.1038/s41467-018-03231-x PMC5840416 29511193

[CIT0009] Hatfield JL, Dold C. 2019. Water-use efficiency: advances and challenges in a changing climate. Frontiers in Plant Science 10, 103. 30838006 10.3389/fpls.2019.00103 PMC6390371

[CIT0010] Hoover DL, Abendroth LJ, Browning DM, et al. 2023. Indicators of water use efficiency across diverse agroecosystems and spatiotemporal scales. Science of the Total Environment 864, 160992. 36535470 10.1016/j.scitotenv.2022.160992

[CIT0011] Leakey ADB, Ferguson JN, Pignon CP, Wu A, Jin Z, Hammer GL, Lobell DB. 2019. Water use efficiency as a constraint and target for improving the resilience and productivity of C_3_ and C_4_ crops. Annual Review of Plant Biology 70, 781–808. 10.1146/annurev-arplant-042817-040305 31035829

[CIT0012] Li XP, Bjorkman O, Shih C, Grossman AR, Rosenquist M, Jansson S, Niyogi KK. 2000. A pigment-binding protein essential for regulation of photosynthetic light harvesting. Nature 403, 391–395. 10667783 10.1038/35000131

[CIT0013] Molden D, Oweis T. 2007. Water for food water for life. A comprehensive assessment of water management in agriculture. Earthscan,4 London.

[CIT0014] Rebetzke GJ, Condon AG, Richards RA, Farquhar GD. 2002. Selection for reduced carbon isotope discrimination increases aerial biomass and grain yield of rainfed bread wheat. Crop Science 42, 739–745.

[CIT0015] Seijger C, Chukalla A, Bremer K, Borghuis G, Christoforidou M, Mul M, Hellegers P, van Halsema G. 2023. Agronomic analysis of WaPOR applications: confirming conservative biomass water productivity in inherent and climatological variance of WaPOR data outputs. Agricultural Systems 211, 103712.

[CIT0016] Steduto P, Hsiao TC, Fereres E. 2007. On the conservative behavior of biomass water productivity. Irrigation Science 25, 189–207.

[CIT0017] Turc B, Sahay S, Haupt J, de Oliveira Santos T, Bai G, Glowacka K. 2024. Up-regulation of non-photochemical quenching improves water use efficiency and reduces whole-plant water consumption under drought. Journal of Experimental Botany 75, 3959–3972. 38470077 10.1093/jxb/erae113 PMC11233411

